# Investigation of Interlaminar Shear Properties of CFRP Composites at Elevated Temperatures Using the Lempel-Ziv Complexity of Acoustic Emission Signals

**DOI:** 10.3390/ma15124252

**Published:** 2022-06-15

**Authors:** Claudia Barile, Caterina Casavola, Giovanni Pappalettera, Vimalathithan Paramsamy Kannan, Gilda Renna

**Affiliations:** Dipartimento di Meccanica, Matematica e Management, Politecnico di Bari, Via Orabona 4, 70125 Bari, Italy; claudia.barile@poliba.it (C.B.); casavola@poliba.it (C.C.); pk.vimalathithan@poliba.it (V.P.K.); gilda.renna@poliba.it (G.R.)

**Keywords:** CFRP, high-temperature applications, Short Beam Shear (SBS) Test, interlaminar shear properties, acoustic emission, SEM micrographs, Lempel-Ziv Complexity, frequency analysis, Continuous Wavelet Transform

## Abstract

Three-point bending tests on Short Beam Shear (SBS) specimens are performed to investigate the interlaminar shear properties of plain weave fabric CFRP composites. The tests are performed in a controlled environmental chamber at two different elevated temperatures. The interlaminar shear properties of the specimens remain largely unaffected by the testing temperature. However, the SEM micrographs show different damage progressions between the specimens tested at 100 °C and 120 °C. Fibre ruptures and longer delamination between the plies, as a result of a high temperature, are observed in the specimens tested at 120 °C, which are not observed in the specimens tested at 100 °C. In addition, the acoustic emission activities during the tests are investigated by using piezoelectric sensors. The information-theoretic parameter, the Lempel-Ziv (LZ) complexity, is calculated for the recorded acoustic signals. The LZ Complexities are used for identifying the occurrence of the first delamination failure in the specimens. Additionally, the two features of the acoustic signals, LZ complexity and Weighted Peak Frequency (W.P-Freq), are used for distinguishing the different damage sources in the CFRP specimens. The results are well-supported by the time-frequency analysis of the acoustic signals using a Continuous Wavelet Transform (CWT).

## 1. Introduction

Non-Destructive Evaluation (NDE) techniques are frequently and adequately used for the structural health monitoring of materials and structures. Despite being used in almost all industrial applications, the debate on the selection of a suitable NDE tool for monitoring and diagnosing composite materials still exists. This is due to the complex damage mechanisms, multiple failure modes and uneven stress distribution in composites. The Acoustic Emission (AE) technique has become one of the most successful NDE tools for monitoring the damage progression and health of composites [1,2,3].

The AE technique is based on the acquisition of stress waves (commonly termed as acoustic waves) by a material when the stored elastic energy is released suddenly. Basically, the AE technique is based on the acquisition and analysis of stress wave signals generated from both internal and external damages occurring in a material. Over the years, researchers have utilised various data processing tools such as pattern recognition algorithms, machine learning algorithms and several signal processing techniques for analysing the acoustic emission signals generated from different types of structural materials [4,5,6,7]. Nonetheless, the necessity for developing new tools and techniques for the comprehensive understanding of acoustic waves and their application in health monitoring is imperative.

Health monitoring or damage diagnostics using the AE technique is proceeded in two ways: a signal-based approach and parameter-based approach. Although the signal-based approach is very efficient, it suffers from the high computational power, storage and (in some cases) the necessity of post-processing the signals [8]. This limits its application in the in-situ health monitoring. Researchers have tried to bridge the gap between the signal-based and parameter-based approach by using AE parameters, which can define the signal-based features of the acoustic waves. For instance, in the field of medical and biomedical applications, several researchers have tried to use information-theoretic parameters such as entropy and complexity to bridge this gap [9,10,11,12]. Some of these parameters, such as Shannon’s entropy, have been used in the health monitoring of rotational components [13,14,15,16,17]. However, the information-theoretic parameters are seldom used in the real-time damage progression or the health monitoring of composites.

In this research work, a complexity parameter, named Lempel-Ziv (LZ) complexity, has been used for monitoring the interlaminar damage progression in Carbon Fibre Reinforced Polymer (CFRP) composites.

CFRP composites are the centre of interest for both the research and industrial sector. Due to their high specific strength and high strength-to-weight ratio, they are preferred in the aerospace and automobile industries. As a result, understanding the mechanical behaviour of the CFRP composites at different environmental conditions, such as elevated temperatures and hygrothermal conditions, is crucial [18,19,20]. Among other mechanical properties of the CFRP, the Interlaminar shear strength (ILSS) is a crucial property of composites in determining their overall performance. This property defines the interfacial adhesion strength of the composites. In most of the studies, the short beam shear (SBS) test is used for investigating the ILSS of the composites. However, it is often debated whether the SBS test can truly estimate the ILSS of the composites or whether a true shear failure occurs in the specimens during SBS tests. Therefore, the investigation of the SBS test in determining the ILSS of the composites using an NDE technique such as AE testing is of paramount importance. Over the years, several researchers have tried to study the ILSS of the composites as the function of specimen size, resin type, fibre type, void content, presence of additives and so on [21,22]. Nonetheless, the effect of temperature on the ILSS of the CFRP composites is seldom studied. It is well-known that the increase in temperature results in the reduction of the off-axis properties of composites [23,24,25,26,27,28,29]. Thus, in this study, the effect of temperature on the ILSS of the CFRP composites is investigated. The AE technique has been used by several researchers in investigating the off-axis properties, such as drop weight impact which has been studied [30,31,32]. Very few researchers have investigated the shear strength of the polymer matrix composites [33]. However, the interlaminar shear properties of the CFRP at elevated temperatures have not been investigated using the AE technique in the literature.

Therefore, this research work aims at investigating the ILSS of the CFRP composites at elevated temperatures. Furthermore, the failure modes and the damage progression in these composites during the SBS tests are proposed to be identified and characterised using the AE technique. The novelty of this research work is the utilisation of LZ complexity in characterising the interlaminar shear properties and identifying the damage modes in CFRP composites.

The goal of this research work can be defined as follows: to investigate the effect of temperature on the ILSS of the CFRP composites; to investigate the failure modes and damage progressions in the CFRP composites during SBS tests; and finally, to investigate the potential of LZ complexity in investigating the damage progressions using the AE technique.

## 2. Materials and Methods

### 2.1. Materials

CFRP specimens with high strength carbon fibres reinforced in plain weave fabric configurations are used for this study. The matrix material is an epoxy resin of density 1.267 g/cm^3^, which constitutes about 41.5% of the prepreg. The carbon fibres used in this study have a high tensile strength of 4900 MPa and tensile modulus of 240 GPa. The fibres have an average density of 1.78 g/cm^3^ in warp and 1.8 g/cm^3^ in weft directions. The prepared prepregs have a high fibre volume content and an average area weight of 192 g/m^2^.

The composites are prepared by stacking up 16 plies of prepregs, having a nominal thickness of 0.218 mm, stacked in the same direction (0° layups). The stacked piles are vacuum bagged with a pressure of 22 inches of Hg and are oven-cured at a temperature of 132 °C for about 120 to 150 min. The glass transition temperature of the composite panels is around 141 °C. Twelve specimens in total are cut from the composite slabs and are used for the SBS tests. The specimens are cut along the layup direction (0°). The span length of the specimens is chosen according to ASTM D2344 standards. The dimensions of the specimen are presented in Table 1. The width and thickness of the specimens are 12.59 mm and 3.53 mm, respectively.

### 2.2. Test Methods

The test setup is essentially a three-point bending test with a loading nose and a pair of supports. The dimensions of the loading nose and the supports are chosen as per ASTM standards. The entire setup is mounted inside an environmental chamber. The tests are conducted at two different temperatures: 100 °C and 120 °C. After mounting the specimens inside the environmental chamber, the temperature is ramped up to the desired level. The specimens are kept ideally inside the chamber at the desired temperature for 5 min before the commencement of testing. The three-point bending load is applied by the crosshead displacement at a speed of 1 mm/min.

**Figure 1:**
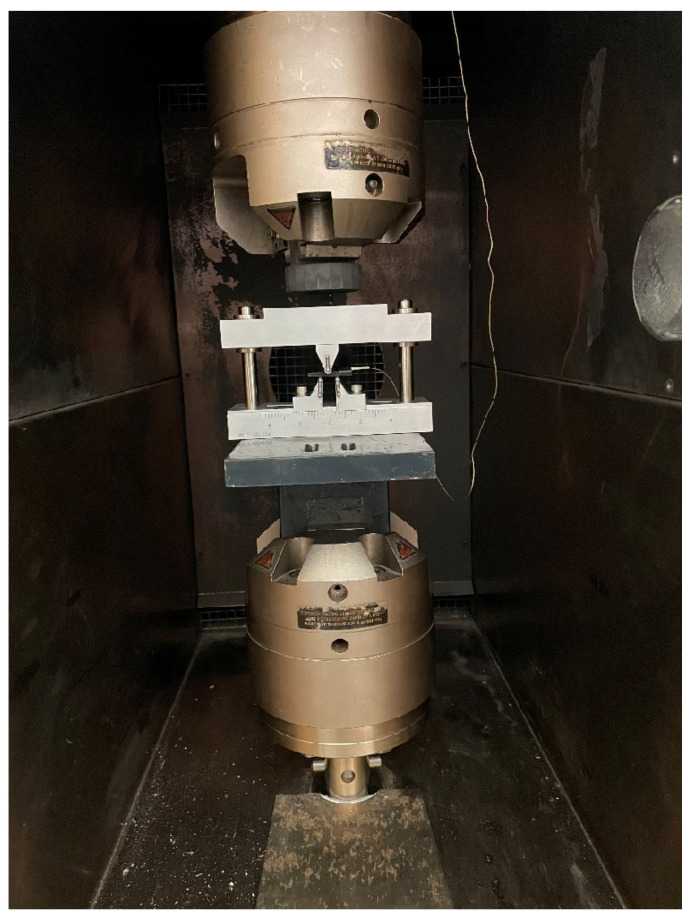
Test Setup showing Short Beam Shear Test Setup with AE sensor inside the environmental chamber.

The test setup also comprises a piezoelectric sensor mounted on top of the specimen for recording the AE signals. The sensor used in this study is PICO sensor (Physical Acoustics Corporation, Princeton Junction, NJ, USA), which has a resonant frequency of 250 kHz and has a high sensitivity to the operating range between 200 kHz to 750 kHz. A thin uniform layer of silicone grease is smeared under the transducing element of the sensor to improve the acoustic coupling. The sensor signals are preamplified by 40 dB and filtered by a low and high band-pass filter of 100 kHz and 1 MHz. Signal waveforms above the detection threshold of 35 dB are recorded at a sampling rate of 2 MHz and 1 k length. For recording each AE event, the Peak Definition Time (PDT), Hit Definition Time (HDT) and Hit Lockout Time (HLT) are set, respectively, as 200 μs, 800 μs and 1000 μs. The test setup with the specimen mounted inside the environmental chamber is presented in Figure 1.

### 2.3. Acoustic Emission Analysis

The recorded AE signals are processed in two ways in this study: parameter-based approach and signal-based approach. Subsequently, these results are compared with the complexity parameter, which will be introduced in the next section. For the parameter-based approach, the AE counts (referred to as counts from herein) are used. Counts are the number of instances the AE signal peaks cross the detection threshold. This parameter has been successfully used by several researchers in identifying the damage initiation or predicting the failure in composites [34,35,36,37,38]. In this work, the preliminary analysis of the AE signals is carried out by comparing the counts with the load–displacement data of the SBS test.

For the signal-based approach, Weighted Peak Frequency (W.P-Freq) is extracted from the recorded signals and is used. It has been evidently shown by many researchers that W.P-Freq of AE signals can potentially be used for identifying different damage sources such as matrix cracking, delamination, or through-thickness crack growth [39,40,41]. In that context, the complexity parameter is compared with the W.P-Freq to test its potential in identifying damage sources. A question may arise why the LZ complexity must be used for damage identification if the same can be achieved by W.P-Freq. Although W.P-Freq can identify the damage sources, it is a frequency-dependent parameter and does not provide any information about the characteristics of the AE signal in the time-frequency domain. Different damage sources in a composite material may generate AE signals whose frequency characteristics may be the same, but may vary in their spectral energy or time-frequency characteristics. One of the goals of this research work is to validate LZ complexity as a sufficient parameter to overcome the limitations of the available AE descriptors for damage characterisation.

For time-frequency analysis of the signals, Continuous Wavelet Transform (CWT) is used. CWT has proved to be an efficient tool in characterising transient signals such as acoustic signals. The feasibility of choosing different wavelets in this type of waveform analysis can extract accurate time-frequency characteristics using the appropriate waveforms. In the authors’ previous studies, a novel method for choosing the appropriate wavelet is developed and discussed [42]. In addition, the procedure for CWT can also be found in the authors’ previous research works and other standard research works [42,43,44,45,46]. To summarise the time-frequency analysis performed in this study, the analytical Morlet wavelet is chosen and used in the CWT.

### 2.4. LZ Complexity

Signal data may have a set of random sequences. These random sequences are due to the presence of frequency harmonics, white noise, or periodic noise in the signal. Complexity is a measure on the extent to which the given sequence resembles a random one. LZ complexity, which was introduced by Lempel and Ziv in 1976, is related to the measure of the number of distinct sequences and the rate of their recurrences in the signal data [47]. In the context of the acoustic signals recorded from different damage sources, the LZ complexity can be defined as the measure of the presence of harmonics or different frequency components and their recurrences in the recorded signal. Based on that, the sources of the acoustic signal (which essentially are different damage sources such as matrix cracking, delamination or fibre breakage) can be identified.

Before calculating the LZ complexity, the signal data must be converted into a finite series with few elements/symbols. In a classical way, the signal data are converted into a binary sequence for simplicity. First, the recorded signal of length n, $S=\left\{s_{1},s_{2},s_{3},\ldots ,\;s_{n}\right\}$ is converted into its analytical form using Hilbert transform [12,14]. The analytical form of the signal is $H=\left\{h_{1},h_{2},\;h_{3},\ldots ,\;h_{n}\right\}$. The analytical form of the signal is then converted into a binary sequence of strings $B=\left\{b_{1},b_{2},\;b_{3},\ldots ,\;b_{n}\right\}$ using the condition in Equation (1).

(1) $$b_{i}=\left\{\begin{array}{c} 0,\;if\;h_{i}<t_{h}\\ 1,\;otherwise \end{array}\right.,$$

where the threshold $t_{h}$ is the median of the analytical signal data H. Once the original signal S is converted into its binary sequence B, the following steps are followed to calculate the LZ complexity.

Step 1: Read the binary sequence B from left to right. Consider *P* and *Q* as two subsequences in *B*. *PQ* is the concatenation of the subsequences *P* and *Q*. Now a deletion operator *χ* is introduced, which deletes the last character of a subsequence. Using the deletion operator on the concatenation *PQ*, one obtains *PQχ*. At the end of first step, after reading the first two strings of $b_{1}$ and $b_{2}$, the complexity counter $c\left(n\right)$ is counted as 1. The other conditions at the end of this step are as follows:

(2) $$c\left(n\right)=1;P=\{b_{1}\};Q=\{b_{2}\};PQ=\{b_{1},b_{2}\};\;∴PQ\pi =\{b_{1}\},$$

Step 2: Read the subsequent strings of *B*. In general, $P=\{b_{1},b_{2},\ldots ,\;b_{r}\}$ and $Q=\{b_{r+1}\}$ and $PQ\chi =\{b_{1},b_{2},\ldots b_{r}\}$. Read the next subsequence $Q_{r+1}$.

Step 3: If the sequence of strings in the subsequence belongs to the set of subsequences *PQχ*, then read the next string $Q_{r+2}$ as $\left\{s_{r+1},\;s_{r+2}\right\}$.

If the subsequence *Q* does not belong to the sequence of strings in the subsequence *PQχ*, then increase the counter of complexity by 1 and nullify the subsequence $Q_{r+2}$; read the next string and take $Q_{r+3}=\left\{b_{r+3}\right\}$.

Step 4: Repeat Step 3 until the whole binary sequence *B* is read.

At the end of Step 4, the complexity counter is normalised to obtain the LZ complexity. This is done by normalising the complexity counter $c\left(n\right)$ with the upper limit of a complexity of a binary string $b\left(n\right)$.

(3) bn=nlog2n,

The LZ complexity LZ is calculated using Equation (4).

(4) LZ=cnbn, 

An example of calculating the LZ complexity using the above steps with a 16-bit binary sequence is provided in Appendix A. For further readings, the readers are directed to the original paper by Lempel and Ziv [47].

### 2.5. SEM Analysis

Morphological characteristics of the specimens before and post SBS tests are analysed using the scanning electron microscopy (SEM) technique to follow the failure evolution. Different damage occurrences are also identified and located. SEM analyses are performed on the mechanically polished cross-section (longitudinal transverse (LT)—short transverse (ST) plane) of the samples using a Zeiss EVO—MA10 scanning electron microscope.

## 3. Results

As mentioned in the introduction section, this research work is aimed at understanding the ILSS of the plain weave fabric CFRP at an elevated temperature, identifying the damage modes and finally validating the capability of LZ complexity in investigating the damage progression. This section is separated to address each of the investigations.

### 3.1. Interlaminar Shear Strength at Elevated Temperatures

The specimens tested at 100 °C and 120 °C, respectively, are named as SBS_100 and SBS_120. Six specimens in each category are tested in the environmental chamber to obtain the maximum shear load at failure and ILSS of the plain weave fabric CFRP composites. The ILSS is calculated using Equation (5), as per the ASTM D2344 standard.

(5) ILSS=0.75 Lmaxbh,

$L_{max}$ is the maximum applied load, b and h are the width and thickness of the specimens.

**Figure 2:**
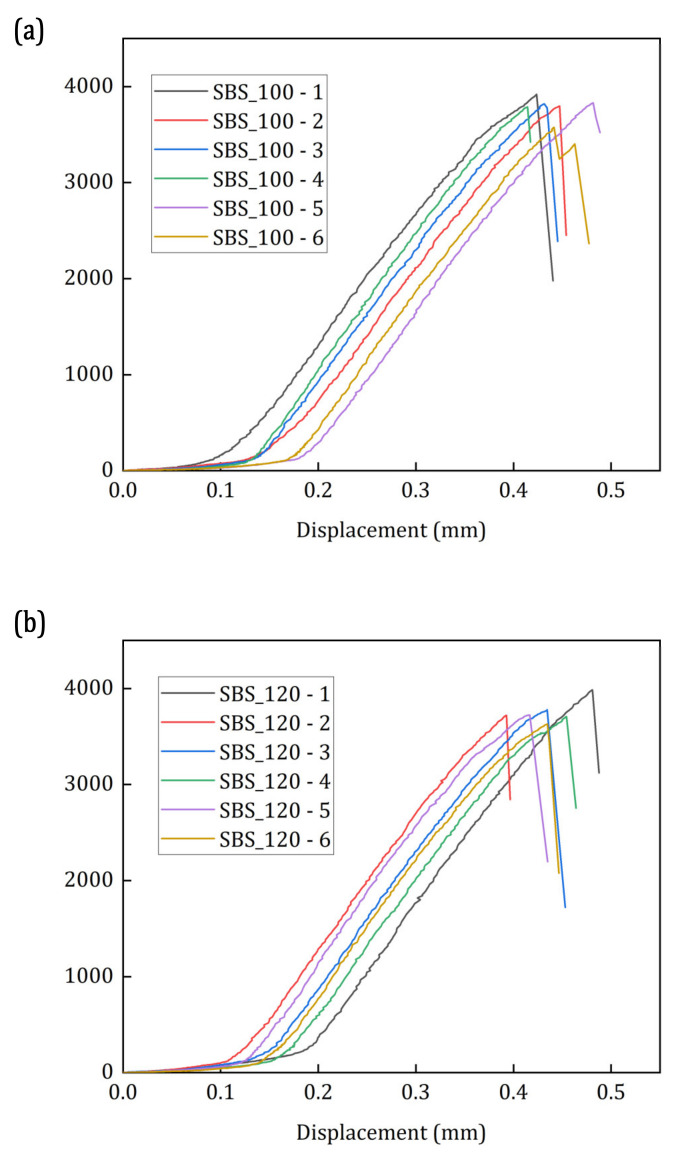
Load–Displacement curves of the SBS specimens tested at (**a**) 100 °C and (**b**) 120 °C.

Figure 2a,b show the load–displacement curves of the SBS tests conducted at 100 °C and 120 °C, respectively. The maximum shear load and the ILSS of the SBS specimens are presented in Table 2.

The load–displacement curves of the two groups of specimens show a linear behaviour before the failure. The specimens fail at 0.4–0.45 mm, without any plastic deformation, visible to the naked eye, before failure. Similar observations can be found in the literature, which indicates a shear failure in the specimens without any apparent signs of compressive damage or large plastic deformation in the specimens [21,22,48]. The results show that the average max. shear load of the specimens tested at 100 °C and 120 °C, respectively, are 3788.92 N and 3758.28 N. There is no significant change in the max. shear load by the increase in test temperature by 20 °C. In fact, the ILSS of the specimens tested at 120 °C is slightly higher than the specimens tested at 100 °C. However, this increase is quite insignificant, and it is safe to say that there is no difference in the ILSS of the two groups of specimens.

It has been noted by several researchers that the interlaminar shear failure in the SBS specimens generally spans across one half of the length of the specimen close to the midplane in the internal layers [21,48]. In the specimens with a larger thickness, the outer plies serve as a protective layer when operated at elevated temperatures. In this case, since the specimens are placed in the high temperatures of 100 °C and 120 °C for only 5 min before the test, the chances of the midplanes being affected by the operating temperature is very slim. This could be the reason for the insignificant changes in the max. shear load and ILSS of the two groups of specimens.

### 3.2. SEM Analysis

The differences in morphological characteristics before and after the SBS tests are further investigated by SEM.

#### 3.2.1. Morphological Characteristics of the SBS Specimens before the Test

**Figure 3:**
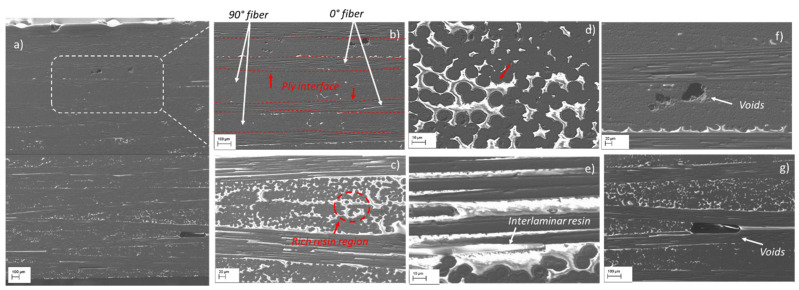
(**a**) SEM images of the mechanically polished cross-section of as-received sample and micrographs at different magnification; (**b**) lay-up configuration [0/90/0]; (**c**) parts of two of the laminae showing the rich-resin region; (**d**) fibres perpendicular (90°) to the plane of the reference; (**e**) fibres along the reference plane (0°); (**f**) small random shaped voids; (**g**) thin void layers (the relatively bright parts are the resin).

Prior to the SBS tests, the microstructure of the as-received samples is analysed to ensure homogeneity. Figure 3a shows a typical SEM image of the mechanically polished cross-section of samples before the test. The SEM micrographs reveal that the microstructure of the sample consists of laminae (plies) of carbon fibres stacked-up and bound together by the epoxy matrix (Figure 3b), with a well-defined interface. The warping fibres in the 0° direction and the 90° direction can be distinctly seen in Figure 3b. The interlaminar interfaces are clearly visible and some of those are highlighted by dashed lines in Figure 3b. Furthermore, the SEM analysis revealed that the microstructure of the samples as-received is rather non-homogeneous and contains evident rich-resin regions both along the interlaminar interfaces and between the fibres perpendicular to the reference plane (Figure 3c–e). In addition to that, the cross-section of the samples exhibits some small discontinuities such as voids (resin-poor regions) and variations in the resin layer thickness between the plane-parallel fibres. Small random shaped voids and thin void layers are also observed (Figure 3f,g). The formation of these poor and rich-resin regions depends on the manufacturing process of the material. The presence of resin-rich regions can affect the static and dynamic mechanical properties as well as the fracture resistance, especially in relation to the temperature [49,50]. Huang et al. have observed that the relatively homogeneous microstructures between the adjacent plies and within the plies, uniform fibre dispersion, fewer resin-rich regions and defects result in a higher ILSS of the composites [51].

#### 3.2.2. Morphological Characteristics of the SBS Specimens Post Failure: Effects of the Temperature

**Figure 4:**
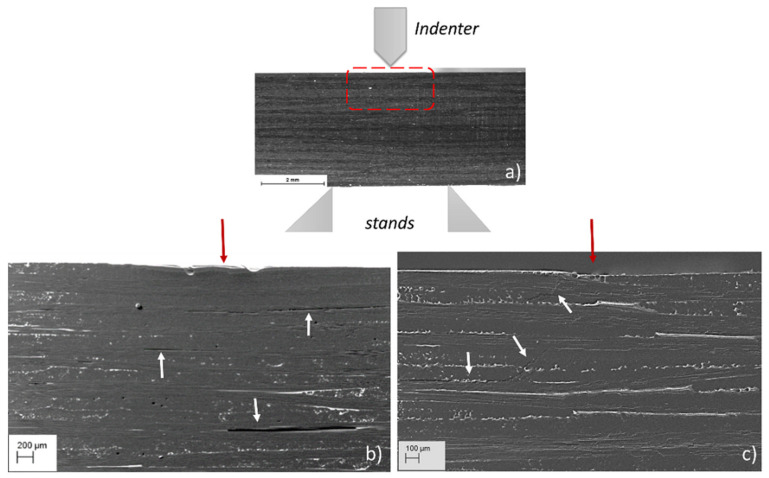
(**a**) Macrograph of the specimen cross-section under investigation and SEM micrographs showing the damage evolution in the compression side of (**b**) SBS_100 and (**c**) SBS_120 specimen after the test. (The red arrows indicate the loading point while the white arrows indicate the extensive damage formed in the specimen cross section).

**Figure 5:**
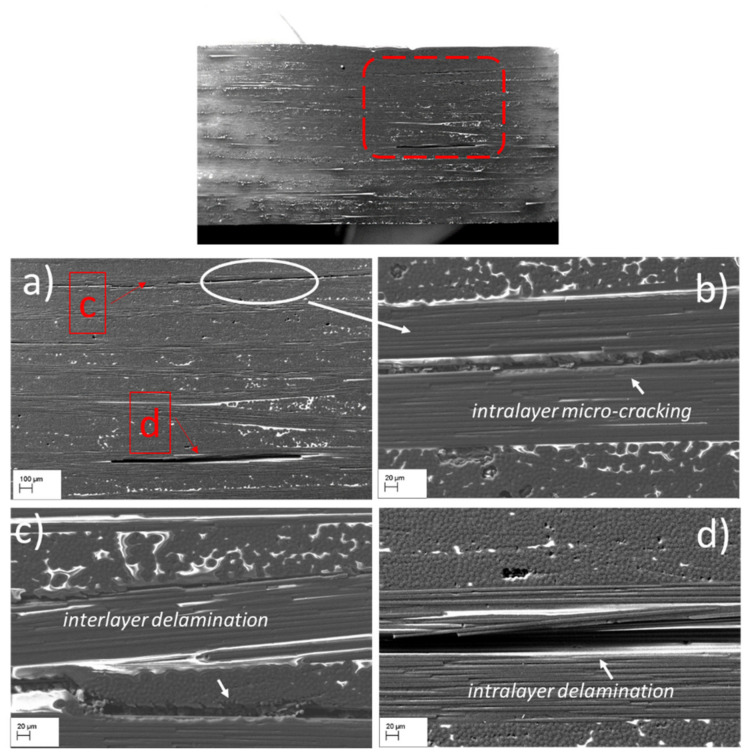
SEM macrograph of the SBS_100 sample cross section: (**a**) enlarged image of the red spot fringe; (**b**) high magnification SEM images showing intralayer micro-cracking; (**c**) high magnification SEM images showing interlaminar delamination; (**d**) high magnification SEM images showing large intralayer delamination between fibre tows parallel to shear plane.

**Figure 6:**
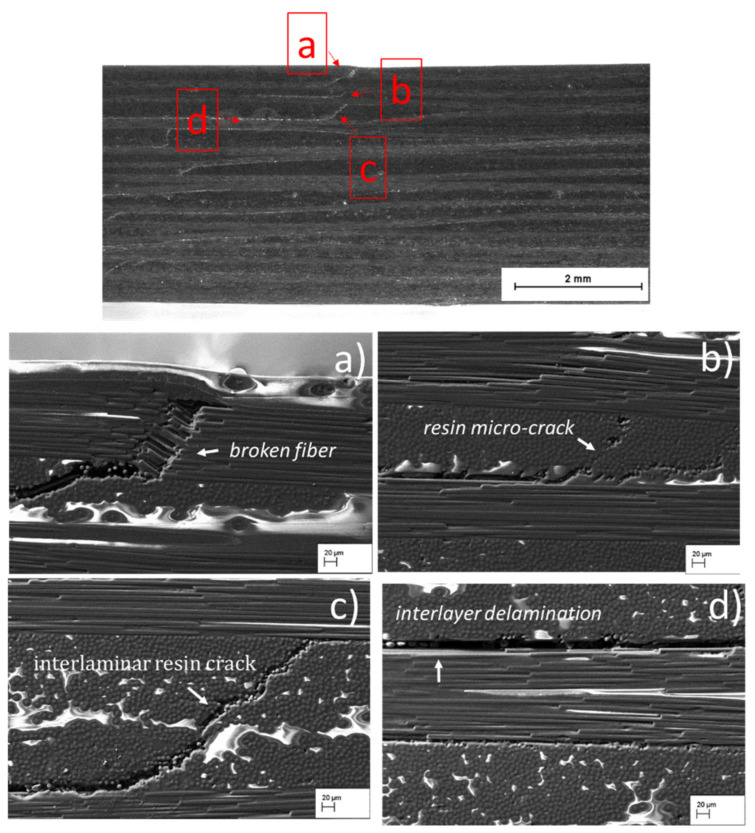
SEM macrograph of the SBS_120 sample cross section and high magnification SEM images of the sample showing some different areas: (**a**) fracture of the first fibres ply under the indenter; (**b**) micro-cracks between the 90° fibres; (**c**) interlaminar cracks along the 90° fibres; (**d**) interlaminar delamination.

After the SBS test, the evolution of bending damage through the thickness of the SBS_100 and SBS_120 composites under the loading point are observed (Figure 4, Figure 5 and Figure 6). Figure 4 depicts an overview of the damage evolution of the internal microstructure in the compression side of the specimen tested at 100 °C compared to the specimen tested at 120 °C. The SEM images reveal that the SBS_100 and SBS_120 specimens did not break, but rather recorded a slight residual deflection. Furthermore, SEM analysis reveals a compressive failure in both SBS specimens, typically occurring as buckling under the loading points, interlaminar damage or through-thickness crack growth through the upper plies. It can be seen that in both samples, defects in the form of fibre/matrix interfacial debonding are dominant and are imaged as dark lines in Figure 4a,b. Extended damages are also indicated by the white arrows (Figure 4).

Although the ILSS measurements (Table 2) do not indicate substantial differences between the two samples, high-magnification the SEM investigations reveal some differences in the damage development. The SEM observations of the SBS_100 sample from the compression and tensile side (Figure 5), reveal the presence only of interlayer delamination (Figure 5a,c) and fibre debonding within in the 0° plies. The initiation of intralayer microcracking was observed in the 7th laminae with 0° fibres orientation (Figure 5b). This microcracking extended along the fibres causing an intralayer delamination until it reached the point of contact between the fibre plies at 0°, after which it continued its path along the interface causing interlaminar debonding (Figure 5c). However, other large delamination at the 18th ply was also observed for this sample (Figure 5a,d).

Instead, the sample tested at 120 °C exhibits both fibre breakage and expanded delamination after the test (Figure 6). In Figure 6a, it can be observed that the load resulted in a fracture of the first fibre ply under the indenter. Particularly, a small portion of the fibre ruptured and assumed an orientation at 45° with respect to 0° fibre ply. Similar to what has been observed in other studies [52,53,54], the sample also showed some very fine micro-cracks starting from the voids (resin-poor regions) present between the 90° fibres (Figure 6b). Moreover, unlike the SBS_100 sample, the presence of large cracks extending along the 90° fibres (Figure 6d), in addition to the delamination (Figure 6c), can be observed in the SBS_120 specimen.

Fibre breakage and the increased delamination observed in this sample can be attributed to the matrix degradation as a result of an increased temperature [49]. In other words, as the temperature rises, the matrix becomes more brittle and, consequently, the overlapping fibres in the warp and weft direction resists severe deformation, which is why the fibres have ruptured under the compressive load.

### 3.3. Acoustic Emission Analysis

**Figure 7:**
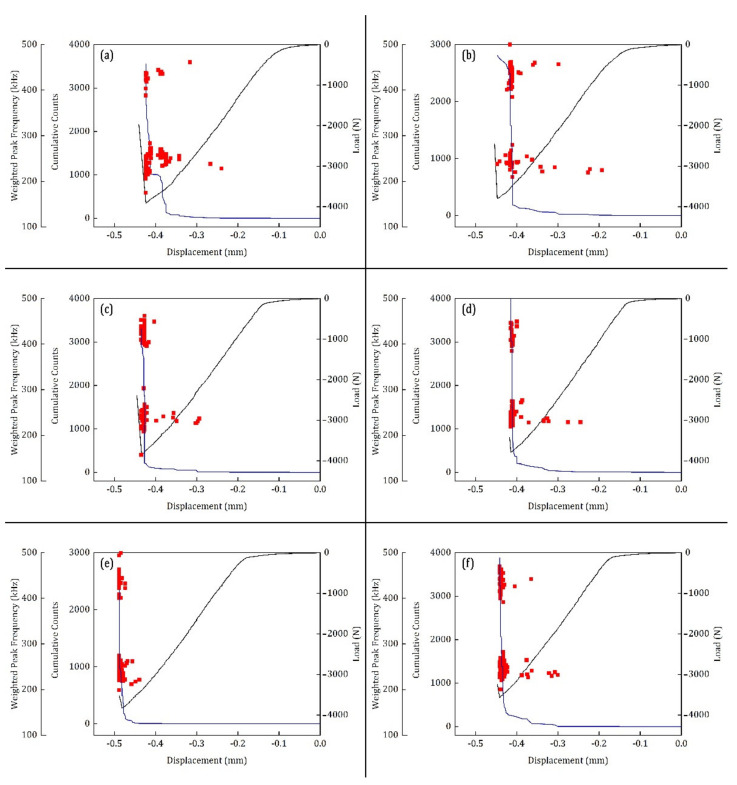
AE descriptors cumulative counts (blue lines) and W.P-Freq (red markers) plotted over the load–displacement curves of the SBS test performed at 100 °C for the CFRP specimens (**a**) SBS_100–1, (**b**) SBS_100–2, (**c**) SBS_100–3, (**d**) SBS_100–4, (**e**) SBS_100–5 and (**f**) SBS_100–6.

**Figure 8:**
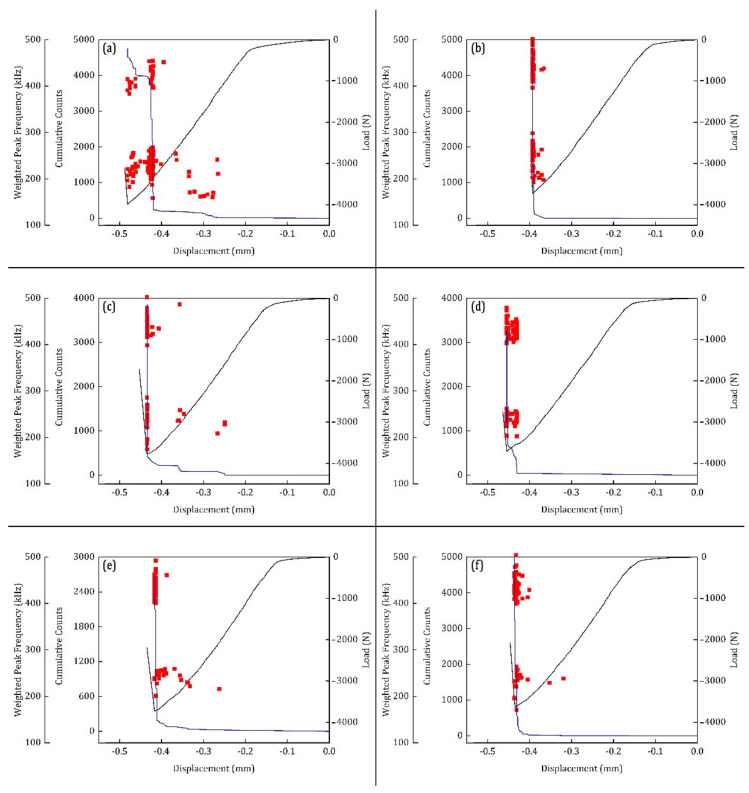
AE descriptors cumulative counts (blue lines) and W.P-Freq (red markers) plotted over the load–displacement curves of the SBS test performed at 100 °C for the CFRP specimens (**a**) SBS_120–1, (**b**) SBS_120–2, (**c**) SBS_120–3, (**d**) SBS_120–4, (**e**) SBS_120–5 and (**f**) SBS_120–6.

The AE parameter, cumulative counts, is used for identifying the major damage occurrences during the SBS tests at two different temperature environments. As mentioned earlier, the counts are the number of instances where the amplitude peak of the recorded AE signal crosses the detection threshold. To identify the extent of difference between the parameter-based descriptors and the signal-based descriptors, the cumulative counts are compared with the W.P-Freq of the recorded signals. The two AE descriptors are plotted over the load–displacement curves of the SBS test. The results of the test conducted at 100 °C and 120 °C are presented in Figure 7 and Figure 8, respectively.

The ILSS properties of the different specimens tested at 100 °C showed apparently less significant differences in Figure 2 and Table 2. However, on closer inspection of Figure 7, some small differences in their properties can be observed. For instance, the first significant damage occurrence in the specimens occurs around the load of 3000 N and a displacement of 0.3 mm in all the specimens, except SBS_100–5 (Figure 7). In SBS_100–5, the first significant damage occurs around 3800 N at a displacement of 0.45 mm (Figure 7f), which is close to the failure. This first significant damage can be identified by a slight increase in the cumulative counts and the appearance of acoustic signals with a W.P-Freq in the 200–300 kHz frequency band. This same behaviour can be found in all the specimens. In the literature, the acoustic signals with a W.P-Freq of 200 kHz–300 kHz is commonly associated with the matrix cracking or delamination event. In the SEM results, matrix cracking can be observed in the specimen in the vicinity of the loading point (Figure 6). In specimen SBS_100–1, the cumulative counts increased in two steps (Figure 7a). First, the cumulative counts increased to 1000 around 3700 N (0.4 mm displacement) and then it increased further closer to failure. This is quite different from the characteristics of the specimens in this group. Upon closer investigation of the load–displacement curves, the slope of the curve changes slightly around 0.4 mm displacement before failure. Similar observations were found by Cui et al. who have found this to be evidence of compressive failure in the SBS specimens [48]. Concurring with this observation, the evidence of compressive failure under the loading points of the SBS specimens are found during SEM analysis (Figure 4).

Apart from this occurrence of first significant damage, the AE signals with W.P-F 200–300 kHz are also generated abundantly closer to the catastrophic failure of the specimen. It is well known that the interlaminar shear failure results in the delamination of the plies. Therefore, these signals can be associated with the delamination.

A second group of AE signals are found predominantly closer to the catastrophic failure of the specimens which have their W.P-Freq above 350 kHz. From the SEM results, it is clear that the major failure mode associated with the SBS specimens is the shear failure (delamination of plies). Nonetheless, there are traces of fibre breakage on the warp direction of the CFRP lamina and some through-thickness crack growth observed in the SEM results (Figure 6). In the literature, the high frequency signals are often associated with fibre breakage and through-thickness crack growth events. Apparently, the W.P-Freq cannot identify the differences between the acoustic signals associated with the fibre breakage and through-thickness crack growth events. Time-frequency analysis of the signals is essential for identifying these different damage modes, which will be explained in the subsequent sections.

The AE descriptor-based results of the SBS specimens tested at 120 °C are presented in Figure 8. Similar to the SBS specimens tested at 100 °C, these specimens also have AE signals in two frequency bands: one between 200 kHz and 300 kHz and the other above 300 kHz. Despite these similarities, only the specimens SBS_120–1, SBS_120–3 and SBS_120–5 (Figure 8a,c,e, respectively) show the first significant damage occurrence around 3000 N. The other three specimens show characteristics similar to SBS_100–5, where the first significance damage occurs close to the failure regime.

Specimen SBS_120–4 shows two steps in the cumulative counts, which is similar to SBS_100–1. The change in the slope of the load response can be found in this specimen in Figure 8d. This is again evidence of the compressive failure. A conclusion can be made from the above observations. When there is a compressive failure in the SBS specimens, a steep increase in cumulative counts can be observed before failure. AE signals recorded from these tests have two frequency characteristics: W.P-Freq between 200 kHz and 300 kHz and W.P-Freq above 350 kHz. The former can be associated with the matrix cracking events or delamination events and the latter can be with the fibre breakage or through-thickness crack growth events. The AE descriptors extracted from the tests can provide a very general discrimination of the different damage modes. However, a more precise classification strategy is required. Therefore, in the next section, the LZ complexity of these signals is used along with the CWT of the signals for a deeper investigation.

### 3.4. LZ Complexity and CWT Results of the Acoustic Emission Signals

**Figure 9:**
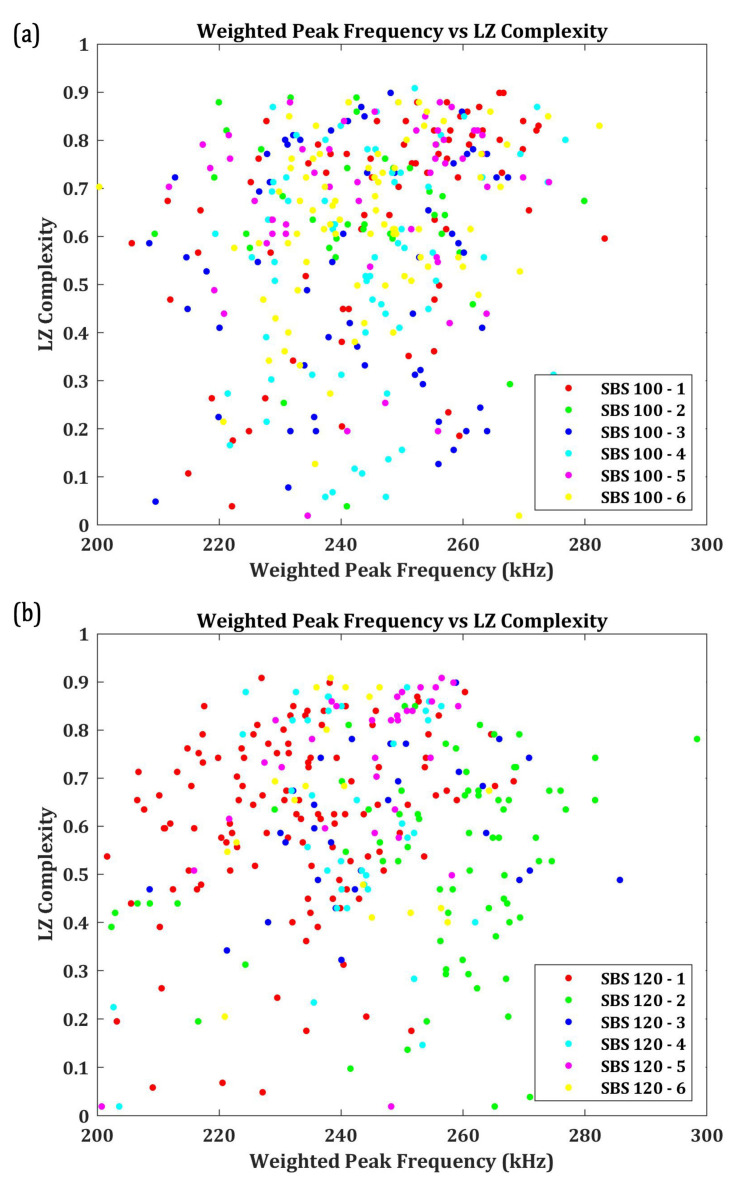
Weighted Peak Frequency (between 200 kHz and 300 kHz) vs. LZ complexity of the AE signals for SBS specimens tested at (**a**) 100 °C and (**b**) 120 °C.

First, the AE signals with the W.P-Freq between 200 kHz and 300 kHz are taken and their LZ complexities are calculated. The extracted signals from SBS specimens tested at 100 °C and 120 °C, respectively, are presented in Figure 9a,b.

**Figure 10:**
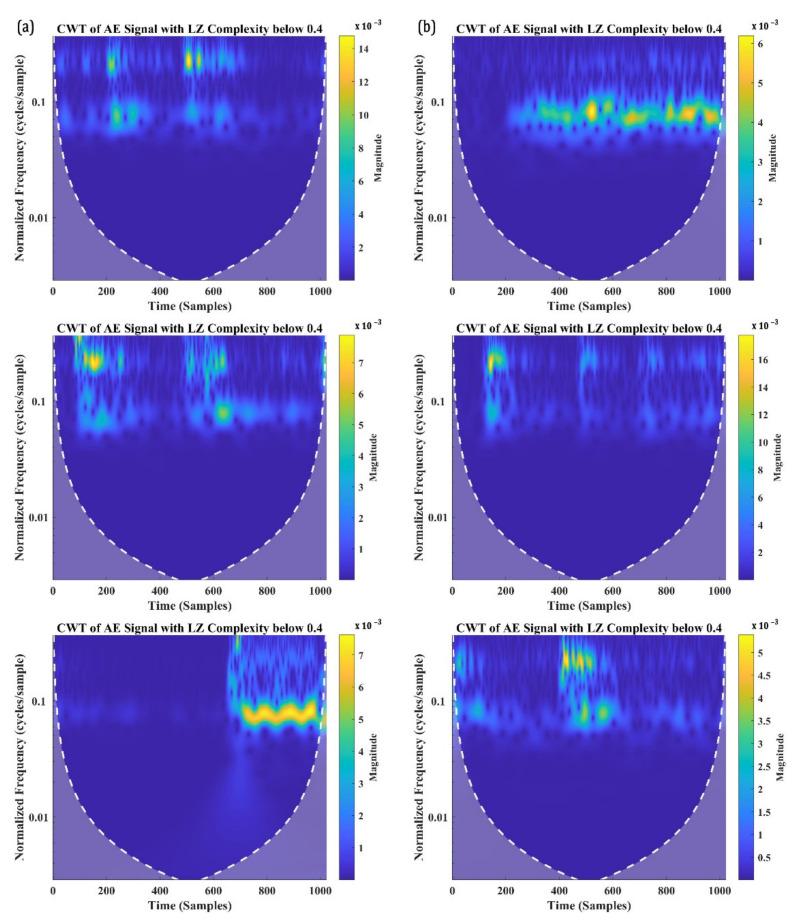
CWT of the AE signals with W.P-Freq between 200 kHz and 300 kHz and LZ complexity below 0.4 recorded from SBS specimens tested at (**a**) 100 °C and (**b**) 120 °C.

**Figure 11:**
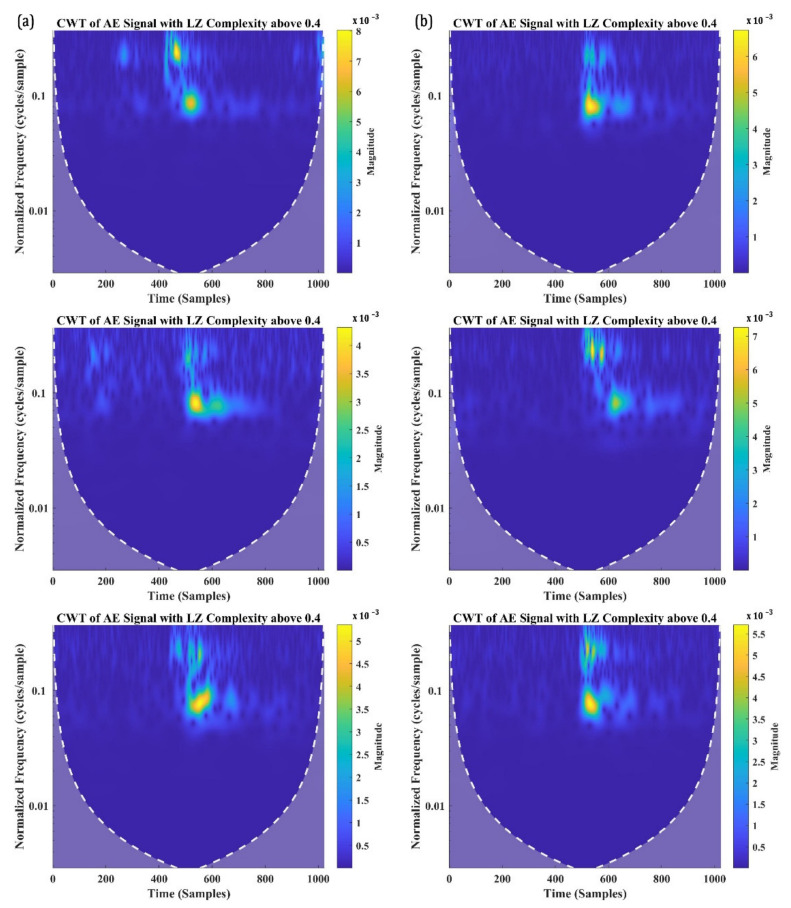
CWT of the AE signals with W.P-Freq between 200 kHz and 300 kHz and LZ complexity above 0.4 recorded from SBS specimens tested at (**a**) 100 °C and (**b**) 120 °C.

Despite that the classification may seem to be randomly distributed, the number of signals with an LZ complexity of 0.4 and above is significantly greater in the distribution. In fact, about 85.64% of signals from SBS_100 and 88.34% of signals from SBS_120 have their LZ complexity above 0.4. To examine these signals further, three signals each with an LZ complexity below 0.4 and above 0.4 are taken and are analysed in their time-frequency domain using CWT. Figure 10a,b shows the CWT of the signals with an LZ complexity below 0.4 from the SBS specimens tested, respectively, at 100 °C and 120 °C. Similarly, the CWT of the AE signals with an LZ complexity above 0.4 is presented in Figure 11a,b.

From Figure 10, it can be seen that the signals having a similar W.P-Freq do not have any similarity in their time-frequency characteristics when their LZ complexity is below 0.4. Bear in mind that these signals constitute less than 15% of the total number of AE signals in the 200 kHz and 300 kHz frequency bands. From their time-frequency characteristics, these are low-frequency signals with a large number of reverberations. During the SBS test, it could be possible that the supporting points in the test setup induced some friction, which caused these noise signals. Another possibility is the friction between the broken matrices or the delaminated layer, which can also generate AE signals with a large number of reverberations. Thus, the AE signals with the W.P-Freq between 200 kHz and 300 kHz and LZ complexity below 0.4 can be deemed as signals from sources such as friction between the broken matrix elements, which cannot be associated with damage or failure modes.

On the other hand, all the AE signals recorded with a W.P-Freq between 200 kHz and 300 kHz and LZ complexity above 0.4 show similar time-frequency characteristics. The similarity in their characteristics are as follows: (a) two frequency bands, one at a normalised frequency of 0.1 cycles/sample and 0.15 cycles/sample; (b) both the frequency bands localised around 500–600 samples; and (c) a similar normalised spectral magnitude—5 to 8 × 10^−3^. AE signals with two localised frequency bands at the same time domain typically indicate their source as delamination in CFRP composites. This concurs with the authors’ previous studies and the other similar literature [8,55,56,57]. Hence, it is safe to say that the AE signals with LZ complexity above 0.4 and W.P-Freq between 200 kHz and 300 kHz are generated from delamination events.

Based on the above observation, the occurrences of the first delamination events are extracted from the SBS tests at 100 °C and 120 °C and are presented in Table 3.

**Figure 12:**
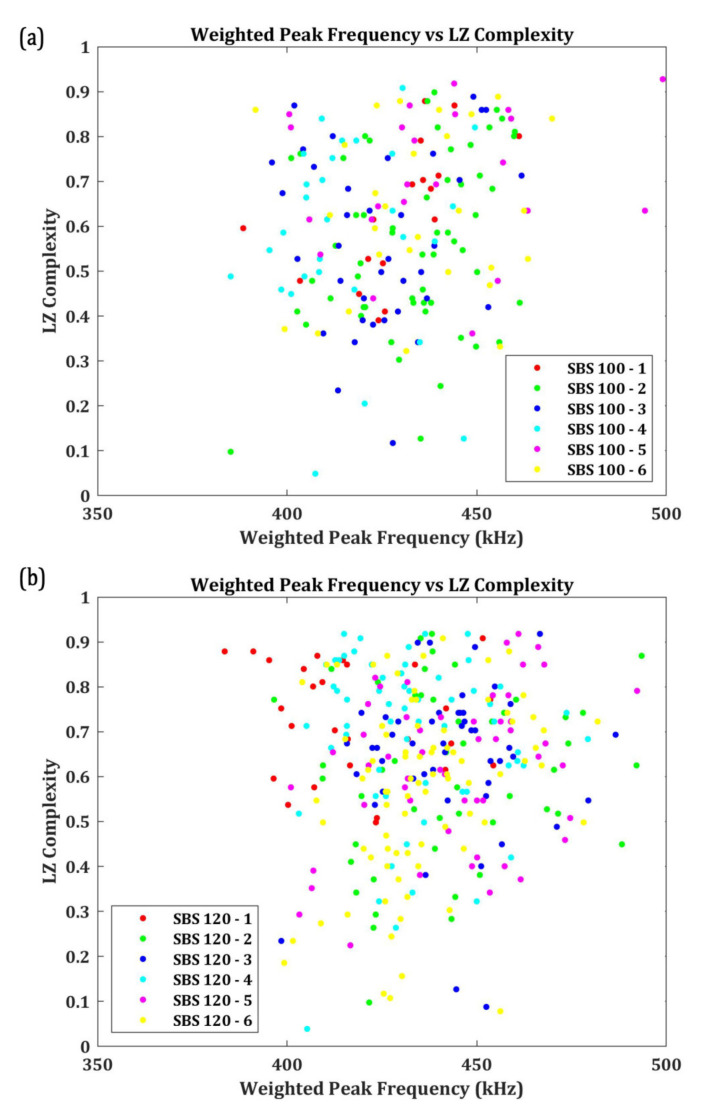
Weighted Peak Frequency (above 350 kHz) vs. LZ complexity of the AE signals for SBS specimens tested at (**a**) 100 °C and (**b**) 120 °C.

LZ complexity of the second group of AE signals, which have a W.P-Freq above 350 kHz are also calculated and their results are presented in Figure 12. Similar to the previous cases, less than 15% of the signals have a W.P-Freq above 350 kHz and LZ complexity less than 0.4.

**Figure 13:**
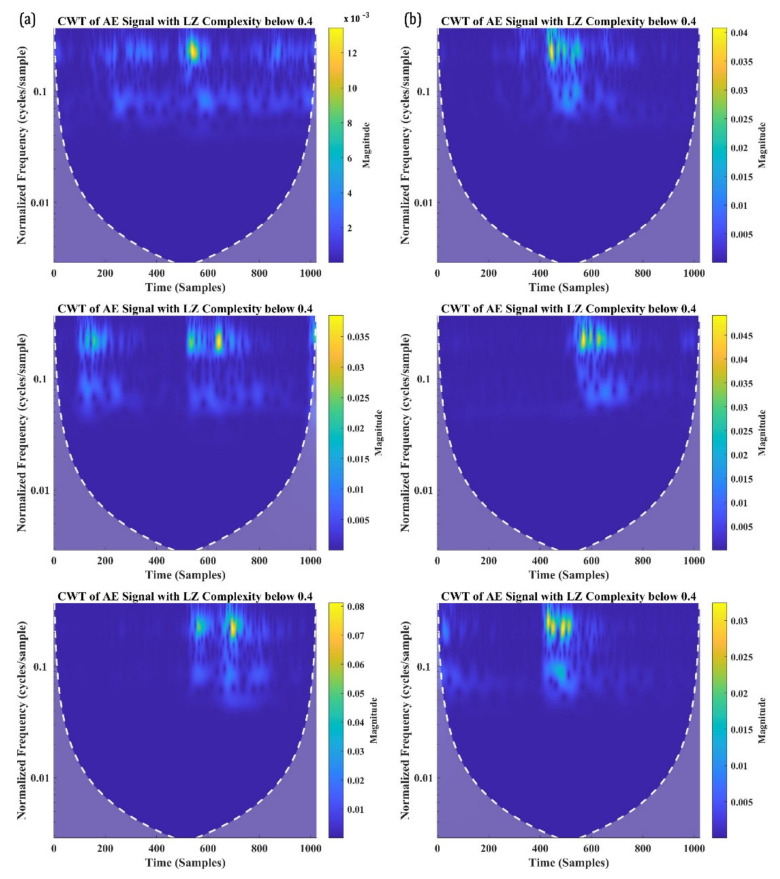
CWT of the AE signals with W.P-Freq above 350 kHz and LZ complexity below 0.4 recorded from SBS specimens tested at (**a**) 100 °C and (**b**) 120 °C.

CWT of the AE signals with a W.P-Freq above 350 kHz and LZ complexity below 0.4 are presented in Figure 13. Figure 13a,b show the CWT of randomly selected signals from SBS tests at 100 °C and 120 °C, respectively. The time-frequency characteristics of all the signals in Figure 13 share similar characteristics, which are explained as follows: (a) one frequency localised at a higher normalised frequency level (which is expected since these signals have a W.P-Freq above 350 kHz), (b) all the signals have a large number of reverberations and (c) they have a considerably larger magnitude compared to the signals shown in Figure 10 and Figure 11. Although these signals have larger reverberations, they cannot be ignored as noise, as considered in the previous group of signals with an LZ complexity below 0.4, because these signals have a significantly higher magnitude and high frequency. Their time-frequency characteristics generally represent the signals generated from through-thickness crack growth in CFRP specimens. It must be noted that these signals constitute less than 15% of the total signals recorded, which makes sense. The through-thickness crack growth is observed in very few signals, originating from the outer ply under the loading point.

**Figure 14:**
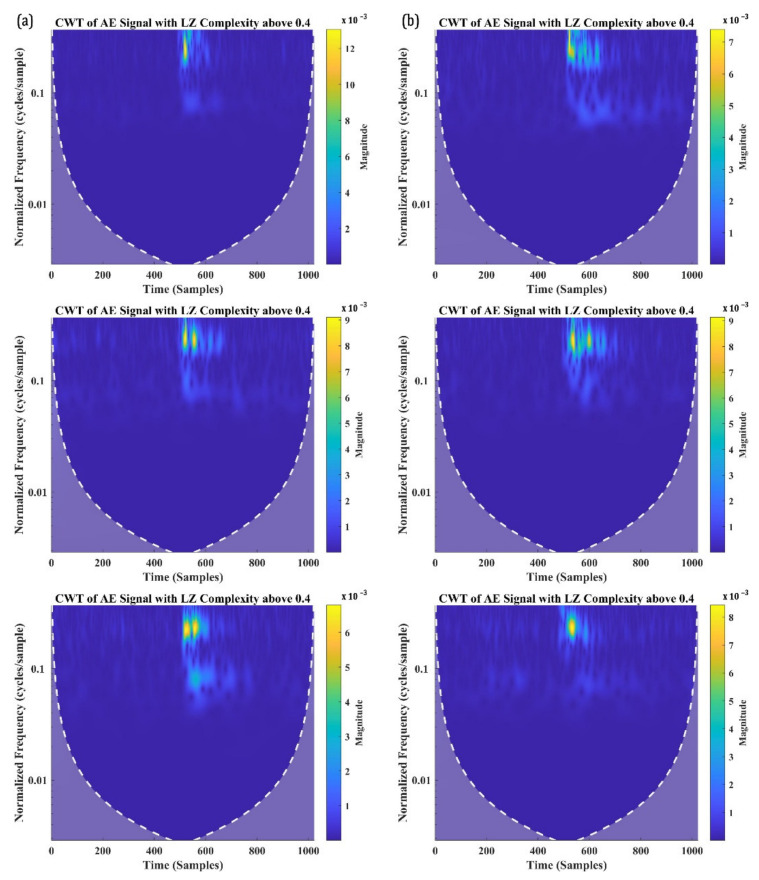
CWT of the AE signals with W.P-Freq above 350 kHz and LZ complexity above 0.4 recorded from SBS specimens tested at (**a**) 100 °C and (**b**) 120 °C.

Finally, the CWT of the AE signals with a W.P-Freq above 350 kHz and LZ complexity greater than 0.4 are presented in Figure 14. These signals also share similar characteristics, which are explained as follows: (a) they have a frequency localised at a higher frequency level, (b) a significantly a smaller number of reverberations compared to the signals in Figure 13 and (c) a significantly smaller spectral magnitude compared to the signals in Figure 13.

These are the characteristics of the acoustic signals generated from both the fibre breakage and through-thickness crack growth of the CFRP specimens. Fibre breakage is observed under the loading point due to the buckling of the overlapping fibres in the warp and weft direction. These possibly could have generated signals with a high W.P-Freq and high LZ complexity.

**Figure 15:**
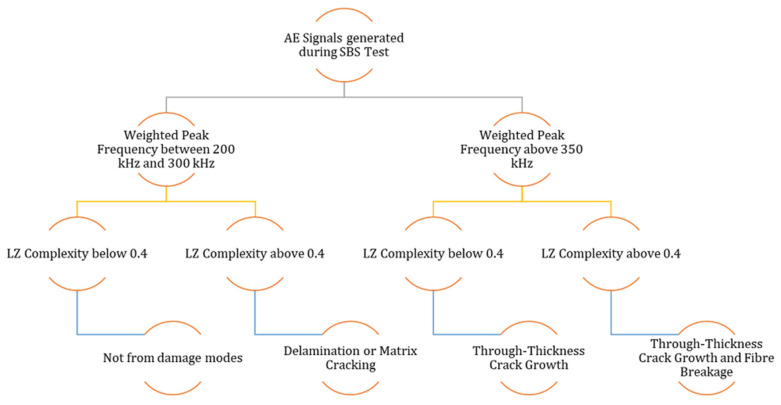
Classification of Damage Modes using W.P-Freq and LZ Complexity.

The classification of damage modes based on the W.P-Freq of the AE signals and their LZ complexity are summarised and presented in Figure 15.

The goal of this research work is to validate LZ complexity as a bridge between the parameter-based analysis and signal-based analysis of AE signals. Evidently, when LZ complexity is used along with W.P-Freq, they have a strong potential to discriminate the AE signals in identifying the damage sources. This can potentially be used for the health monitoring of the composites. It has been argued by several researchers over the years that using a solitary parameter for damage analysis using the AE technique is rather inefficient. In that regard, the potential of the LZ complexity with W.P-Freq can open the possibility of efficient damage characterisation.

## 4. Conclusions

CFRP specimens with fibres oriented in plain weave fabric are tested in this study at two different temperatures. SEM micrographs of the specimens before testing reveal evident non-homogeneity, resin-rich regions between the interlaminar interfaces and the warp and weft direction of the fibres. In addition, small random shaped voids are observed in some regions, which could affect the ILSS of the CFRP specimens. Nonetheless, the ILSS properties of the CFRP specimens from the SBS test do not show any significant changes due to the increase in the testing temperature. However, SEM micrographs of the specimens post-failure show differences in damage modes in the specimens tested at two different temperatures. Both SBS_100 and SBS_120 show compressive failure under the loading point. The dominant failure remains interfacial debonding. However, SBS_100 shows only interplay debonding and fibre debonding. However, SBS_120 also exhibits fibre ruptures, which possibly is due to the increase in testing temperature.

The AE technique is used for identifying the damage occurrences in the test specimens. The cumulative counts of the AE signals are able to identify the occurrence of compressive failure in the specimens. A new parameter, named LZ complexity, is introduced for identifying the different damage modes. When this parameter is used with the W.P-Freq of the AE signals, it has the potential to identify the damage evolution in the CFRP specimens. The recorded AE signals are separated into four groups based on their W.P-Freq and LZ complexity values in an attempt to identify the damage modes.

1. AE signals with a W.P-Freq between 200 kHz and 300 kHz and LZ complexity below 0.4 are mostly signals from sources other than failure modes such as friction between broken matrix elements
2. AE signals with a W.P-Freq between 200 kHz and 300 kHz and LZ complexity greater than 0.4 are predominantly from delamination events and matrix cracking events
3. AE signals with a W.P-Freq above 350 kHz and LZ complexity below 0.4 are from through-thickness crack growth
4. AE signals with a W.P-Freq above 350 kHz and LZ complexity greater than 0.4 are apparently from through-thickness crack growth and fibre breakage

In addition to the identification of first delamination damage, the LZ complexity also shows its capability in categorising the acoustic emission signals generated from different damage modes. This is made possible without the utilisation of time-consuming post-processing of data. However, the LZ complexity cannot identify different damage modes independently. It must be supported by the W.P-Freq of the AE signals. Nonetheless, it has the potential to bridge the gap between the parameter-based and signal-based approaches of using the AE technique. This paves way for the introduction of other information-theoretic parameters in the health monitoring of composite materials and structures.

## Figures and Tables

**Table 1:** Dimensions of the SBS Specimens tested at 100 °C and 120 °C.

Specimen Name	Width	Thickness
	mm	mm
Specimens tested at 100 °C		
SBS_100–1	12.65	3.54
SBS_100–2	12.56	3.59
SBS_100–3	12.52	3.54
SBS_100–4	12.62	3.57
SBS_100–5	12.63	3.54
SBS_100–6	12.63	3.51
Specimens tested at 120 °C		
SBS_120–1	12.65	3.55
SBS_120–2	12.57	3.54
SBS_120–3	12.62	3.48
SBS_120–4	12.62	3.55
SBS_120–5	12.58	3.59
SBS_120–6	12.65	3.56

**Table 2:** ILSS Properties of Plain Weave Fabric CFRP specimens tested at 100 °C and 120 °C.

Specimen Name	Max. Shear Load	ILSS
	N	MPa
SBS_100–1	3919	65.19
SBS_100–2	3797	64.27
SBS_100–3	3821	63.62
SBS_100–4	3788	63.54
SBS_100–5	3831	64.83
SBS_100–6	3574	61.91
**Average**	**3788.33**	**62.82**
*Std.dev*	*114.87*	*3.04*
SBS_120–1	3986	66.58
SBS_120–2	3722	62.74
SBS_120–3	3779	64.55
SBS_120–4	3708	62.08
SBS_120–5	3722	61.83
SBS_120–6	3630	60.46
**Average**	**3757.83**	**63.04**
*Std.dev*	*121.57*	*2.19*

**Table 3:** Load at First Delamination Failure in SBS specimens based on the W.P-Freq and LZ Complexity.

Specimen Name	Load at First Delamination	Specimen Name	Load at First Delamination
	N		N
SBS_100–1	1889	SBS_120–1	1274
SBS_100–2	1089	SBS_120–2	3518
SBS_100–3	2206	SBS_120–3	1586
SBS_100–4	1681	SBS_120–4	3535
SBS_100–5	3444	SBS_120–5	3022
SBS_100–6	2153	SBS_120–6	2475

## Data Availability

The data used in this research work cannot be shared publicly. They can be obtained by contacting the corresponding author.

## Appendix A

Calculation procedure for the Lempel-Ziv complexity of a string. Consider a finite binary string of a finite length (n=16), $B=\left\{1010010110101001\right\}$.

Initially,

$$\begin{array}{l} i=0\;\left|PQ\chi_{1}=\left\{\;\right\}\;\right|\;Q_{0}=\left\{\;\right\}\;|\;c\left(n\right)=0\\ i=1\;\left|PQ\chi_{1}=\left\{1\right\}\;\right|\;Q_{1}=\left\{1\right\}\;\left|Q_{1}\notin PQ\chi_{1}\to c\left(n\right)=1\;\right|\;Q_{1}=\left\{\;\right\}\\ i=2\;\left|PQ\chi_{2}=\left\{10\right\}\;\right|\;Q_{2}=\left\{0\right\}\;\left|Q_{2}\notin PQ\chi_{2}\to c\left(n\right)=2\;\right|\;Q_{2}=\left\{\;\right\}\\ i=3\;\left|PQ\chi_{3}=\left\{101\right\}\;\right|\;Q_{3}=\left\{1\right\}\;\left|Q_{3}\in PQ\chi_{3}\to c\left(n\right)=2\;\right|\;Q_{3}=\left\{1\right\}\\ i=4\;\left|PQ\chi_{4}=\left\{1010\right\}\;\right|\;Q_{4}=\left\{10\right\}\;\left|Q_{4}\in PQ\chi_{4}\to c\left(n\right)=2\;\right|\;Q_{4}=\left\{10\right\}\\ i=5\;\left|PQ\chi_{5}=\left\{10100\right\}\;\right|\;Q_{5}=\left\{100\right\}\;\left|Q_{5}\notin PQ\chi_{5}\to c\left(n\right)=3\;\right|\;Q_{5}=\left\{\;\right\}\\ i=6\;\left|PQ\chi_{6}=\left\{101001\right\}\;\right|\;Q_{6}=\left\{1\right\}\;\left|Q_{6}\in PQ\chi_{6}\to c\left(n\right)=3\;\right|\;Q_{6}=\left\{1\right\}\\ i=7\;\left|PQ\chi_{7}=\left\{1010010\right\}\;\right|\;Q_{7}=\left\{10\right\}\;\left|Q_{7}\in PQ\chi_{7}\to c\left(n\right)=3\;\right|\;Q_{7}=\left\{10\right\}\\ i=8\;\left|PQ\chi_{8}=\left\{10100101\right\}\;\right|\;Q_{8}=\left\{101\right\}\;\left|Q_{8}\in PQ\chi_{8}\to c\left(n\right)=3\;\right|\;Q_{8}=\left\{101\right\}\\ i=9\;\left|PQ\chi_{9}=\left\{101001011\right\}\;\right|\;Q_{9}=\left\{1011\right\}\;\left|Q_{9}\notin PQ\chi_{9}\to c\left(n\right)=4\;\right|\;Q_{9}=\left\{\;\right\}\\ i=10\;\left|PQ\chi_{10}=\left\{1010010110\right\}\;\right|\;Q_{10}=\left\{0\right\}\;\left|Q_{10}\in PQ\chi_{10}\to c\left(n\right)=4\;\right|\;Q_{10}=\left\{0\right\}\\ i=11\;\left|PQ\chi_{11}=\left\{10100101101\right\}\;\right|\;Q_{11}=\left\{01\right\}\;\left|Q_{11}\in PQ\chi_{11}\to c\left(n\right)=4\;\right|\;Q_{11}=\left\{01\right\}\\ i=12\;\left|PQ\chi_{12}=\left\{101001011010\right\}\;\right|\;Q_{12}=\left\{010\right\}\;\left|Q_{12}\in PQ\chi_{12}\to c\left(n\right)=4\;\right|\;Q_{12}=\left\{010\right\}\\ i=13\;\left|PQ\chi_{13}=\left\{1010010110101\right\}\;\right|\;Q_{13}=\left\{0101\right\}\;\left|Q_{13}\in PQ\chi_{13}\to c\left(n\right)=4\;\right|\;\;Q_{13}=\left\{0101\right\}\\ i=14\;\left|PQ\chi_{14}=\left\{10100101101010\right\}\;\right|\;Q_{14}=\left\{01010\right\}\;\left|Q_{14}\notin PQ\chi_{14}\to c\left(n\right)=5\;\right|\;Q_{14}=\left\{\;\right\}\\ i=15\;\left|PQ\chi_{15}=\left\{101001011010100\right\}\;\right|\;Q_{15}=\left\{0\right\}\;\left|Q_{15}\in PQ\chi_{15}\to c\left(n\right)=5\;\right|\;Q_{15}=\left\{0\right\}\\ i=16\;\left|PQ\chi_{16}=\left\{1010010110101001\right\}\;\right|\;Q_{16}=\left\{01\right\}\;\left|Q_{16}\in PQ\chi_{16}\to c\left(n\right)=5\;\right|\;Q_{16}=\left\{01\right\} \end{array}$$

At the end of the sequence, the complexity counter $c\left(n\right)=5$. Now, using Equation (5), the $b\left(n\right)$ can be calculated as 4. Now the LZ complexity can be calculated by Equation (4). The LZ complexity for the sequence B is 1.25.
